# Co-occurrence of hearing loss and posttraumatic stress disorder among injured military personnel: a retrospective study

**DOI:** 10.1186/s12889-020-08999-6

**Published:** 2020-07-08

**Authors:** Andrew J. MacGregor, Antony R. Joseph, G. Jay Walker, Amber L. Dougherty

**Affiliations:** 1 Medical Modeling, Simulation, and Mission Support Department, Naval Health Research Center, 140 Sylvester Road, San Diego, CA USA; 2 Hearing Loss Prevention Laboratory, Communication Sciences and Disorders Department, Illinois State University, Normal, IL USA; 3 Leidos, Inc., San Diego, CA USA

**Keywords:** Hearing loss, PTSD, Military, Veteran

## Abstract

**Background:**

Posttraumatic stress disorder (PTSD) and hearing loss are hallmark public health issues related to military service in Iraq and Afghanistan. Although both are significant individual contributors to disability among veterans, their co-occurrence has not been specifically explored.

**Methods:**

A total of 1179 male U.S. military personnel who sustained an injury between 2004 and 2012 during operations in Iraq or Afghanistan were identified from clinical records. Pre- and postinjury audiometric data were used to define new-onset hearing loss, which was categorized as unilateral or bilateral. Diagnosed PTSD was abstracted from electronic medical records. Logistic regression analysis examined the relationship between hearing loss and PTSD, while adjusting for age, year of injury, occupation, injury severity, injury mechanism, and presence of concussion.

**Results:**

The majority of the study sample were aged 18–25 years (79.9%) and sustained mild-moderate injuries (94.6%). New-onset hearing loss was present in 14.4% of casualties (10.3% unilateral, 4.1% bilateral). Rates of diagnosed PTSD were 9.1, 13.9, and 29.2% for those with no hearing loss, unilateral hearing loss, and bilateral hearing loss, respectively. After adjusting for covariates, those with bilateral hearing loss had nearly three-times higher odds of PTSD (odds ratio = 2.92; 95% CI, 1.47–5.81) compared to those with no hearing loss. Unilateral hearing loss was not associated with PTSD.

**Conclusions:**

Both PTSD and hearing loss are frequent consequences of modern warfare that adversely affect the overall health of the military. Bilateral, but not unilateral, hearing loss was associated with a greater burden of PTSD. This has implications for warfighter rehabilitation and should encourage collaboration between audiology and mental health professionals.

## Background

Posttraumatic stress disorder (PTSD) is a frequent wartime problem [1–3]. The disorder is characterized by anxiety, detachment, avoidance behaviors, and intrusive thoughts in response to a traumatic event [4]. The post-9/11 military conflicts in Iraq and Afghanistan produced a significant PTSD health burden, resulting in increased rates of hospitalization, a surge in disability claims, and an array of comorbid physical and mental health conditions [5–9]. With military members eventually transitioning to civilian life, the effects of PTSD become a wider public health issue [10].

Similar to PTSD, hearing loss is a significant public health problem in the military, as the Department of Veterans Affairs (VA) reported that the cost of hearing loss exceeds one billion dollars annually [11]. In general, military personnel have a higher risk of hearing loss than their civilian counterparts [12]. During a military wartime deployment, there are multiple risk factors for hearing loss, including repeated occupational noise exposures as well as blast exposure [13]. Asymmetrical warfare employed by the enemy was prevalent in Iraq and Afghanistan (e.g., improvised explosive devices), which resulted in blasts causing the majority of all injuries [14, 15]. It is well established that exposure to blasts leads to adverse effects on the auditory system [16–19]. A recent retrospective cohort study found a more than two-fold increase in risk of hearing loss among military personnel with blast relative to nonblast injuries [19].

Although PTSD and hearing loss both contribute significantly to the overall health burden in the military, their co-occurrence has not been widely described in the literature [20]. A VA study by Swan and colleagues was the first to suggest the association, finding that those with a diagnosis of hearing loss were more likely to have a diagnosis of PTSD [21]. This study used diagnostic codes to identify hearing loss and was unable to achieve the level of granularity found with audiometric data. One civilian study found a higher rate of anxiety disorder in a community sample with sensorineural hearing loss compared with controls, but PTSD was not specifically explored [22]. Further, hearing loss and PTSD have been individually linked to physical injury [19, 23], most notably blast-related concussion, a novel injury type of increased military importance over the course of the Iraq and Afghanistan conflicts [24, 25].

The paucity of literature on hearing loss and PTSD, in addition to the combined public health impacts, warrants an investigation of their co-occurrence. The present study aimed to examine the relationship between new-onset hearing loss and PTSD, as measured using audiometric data, among a sample of U.S. military personnel with deployment-related injury. It was hypothesized that hearing loss would be positively associated with PTSD diagnosis.

## Methods

### Study sample

Military personnel injured during combat deployment in Iraq and Afghanistan between 2004 and 2012 were identified from the Expeditionary Medical Encounter Database (EMED), which contains clinical encounters across all levels of medical care [26]. The EMED clinical record has provider notes and information that describe the injury event, including mechanism and severity, which is then verified by certified nurse coders. Data extracted from the EMED can be connected with other Department of Defense (DoD) medical databases (e.g., Military Health System Data Repository [MDR]), as well as tactical, personnel, operational, and deployment-related data.

A subset of the EMED was used to create the Blast-Related Auditory Injury Database (BRAID), which includes injured military personnel, specifically Navy and Marine Corps, with valid audiometric information from the DoD Hearing Conservation Program [27]. Details regarding the creation of the BRAID from the EMED have been published elsewhere [18]. The BRAID includes service members with blast- and nonblast-related injuries sustained during deployment. Due to small numbers of women in the BRAID, only men were included in this analysis. For inclusion, per previous research on the BRAID, individuals in the present study had audiometric data within 1 year preinjury and 1 year postinjury (*n* = 1573). After excluding those with a previous PTSD diagnosis (*n* = 6) and those with preinjury hearing loss (*n* = 388), the final study sample consisted of 1179 individuals. This study received local Institutional Review Board approval.

### Variables and measures

#### Demographic characteristics

Included age at time of injury, which was calculated as the difference between date of injury and birth and categorized as 18–25 or 26 and older. Occupation was dichotomized per DoD Military Occupational Specialty code into infantry or non-infantry [28].

#### Injury-related characteristics

Were abstracted from the EMED clinical record. Year of injury was categorized as 2004–2006 or 2007–2012. Battle injury was categorized as battle or nonbattle, and blast injury as blast or nonblast. Injuries were distinguished by battle and blast injury in order to account for combat noise exposure. Injury severity was measured using the Injury Severity Score (ISS), which provides an overall measure of injury severity for individuals with multiple injuries [29, 30]. The ISS is calculated using Abbreviated Injury Scale scores [31] ranging from 0 (*no injury*) to 75 (*unsurvivable injury*). In the present study, ISS was categorized as 1–8 (i.e., mild-moderate injury severity) or 9 and greater (i.e., serious injury and worse) [29–31]. Concussion was indicated if documented in the EMED clinical record at the time of injury.

#### Hearing loss

Was measured using audiometric pure-tone hearing threshold levels for left and right ears at six test frequencies (500 Hz, 1000 Hz, 2000 Hz, 3000 Hz, 4000 Hz, and 6000 Hz) that were abstracted from the BRAID. Postinjury hearing loss was defined as a response exceeding 25 dB at any test frequency [19]. Postinjury hearing loss was further categorized into unilateral if present in only one ear or bilateral if present in both ears.

#### PTSD

Diagnosis was obtained from outpatient medical information in the MDR. PTSD was defined as presence of an International Classification of Diseases, 9th Revision, Clinical Modification code of 309.81 within 2 years postinjury [32].

### Statistical analyses

Data management and statistical analyses were performed using SAS software, version 9.4 (SAS Institute, Inc., Cary, North Carolina). Characteristics of the study sample were stratified by hearing loss status and chi-square statistics were used to test for differences. Tetrachoric correlation coefficients were calculated to highlight relationships between covariates. Univariate and multivariate logistic regression with backward selection was used to model the relationship between hearing loss and PTSD. The Hosmer-Lemeshow test was used to assess the model for goodness of fit with an alpha level of 0.10.

## Results

Overall, unilateral and bilateral hearing loss was present in 10.3% (*n* = 122) and 4.1% (*n* = 48) of study participants, respectively. Sample characteristics are shown in Table 1. The majority were aged 18–25 years at time of injury, in non-infantry positions, and injured between the years 2004 and 2006. Nonblast and nonbattle injuries predominated, and concussion was present in 26.2% of personnel. Among those with bilateral hearing loss, 72.9% were injured by a blast compared with only 37.5% for those with no hearing loss. Rates of hearing loss were significantly higher in infantry personnel (*p* = 0.003), and those with battle injury (*p* < 0.001), blast injury (*p* < 0.001), and concussion (*p* = 0.002). Hearing loss groups did not statistically differ by age or ISS (*p*s > 0.05). The overall rate of PTSD was 10.4%, and rates differed significantly across hearing loss groups (*p* < 0.001). Those with bilateral hearing loss had the highest PTSD rate at 29.2%, followed by unilateral hearing loss at 13.9%, and no hearing loss at 9.1%.

**Table 1:** Descriptive characteristics of the study sample

Variable	Total No. (%) (*n* = 1179)	No hearing loss No. (%) (*n* = 1009)	Unilateral hearing loss No. (%) (*n* = 122)	Bilateral hearing loss No. (%) (*n* = 48)	*p*-value
Age, years					0.793
18–25	942 (79.9)	806 (79.9)	96 (78.7)	40 (83.3)	
26 and older	237 (20.1)	203 (20.1)	26 (21.3)	8 (16.7)	
Year of injury					< 0.001
2004–2006	765 (64.9)	677 (67.1)	67 (54.9)	21 (43.8)	
2007–2012	414 (35.1)	332 (32.9)	55 (45.1)	27 (56.3)	
Occupation					0.003
Non-infantry	603 (51.2)	536 (53.1)	46 (37.7)	21 (43.8)	
Infantry	576 (48.9)	473 (46.9)	76 (62.3)	27 (56.3)	
Battle injury					< 0.001
No	614 (52.1)	557 (55.2)	44 (36.1)	13 (27.1)	
Yes	565 (47.9)	452 (44.8)	78 (63.9)	35 (72.9)	
Blast injury					< 0.001
No	699 (59.3)	631 (62.5)	55 (45.1)	13 (27.1)	
Yes	480 (40.7)	378 (37.5)	67 (54.9)	35 (72.9)	
ISS					0.647
1–9	1115 (94.6)	956 (94.8)	115 (94.3)	44 (91.7)	
9 and greater	64 (5.4)	53 (5.3)	7 (5.7)	4 (8.3)	
Concussion					0.002
No	870 (73.8)	760 (75.3)	84 (68.9)	26 (54.2)	
Yes	309 (26.2)	249 (24.7)	38 (31.2)	22 (45.8)	
PTSD					< 0.001
No	1056 (89.6)	917 (90.9)	105 (86.1)	34 (70.8)	
Yes	123 (10.4)	92 (9.1)	17 (13.9)	14 (29.2)	

*ISS* Injury Severity Score, *PTSD* posttraumatic stress disorder

The tetrachoric correlation matrix for covariates is shown in Table 2. The highest correlations were between battle injury and blast injury (0.994), blast injury and concussion (0.851), and battle injury and concussion (0.798). Notably, only one nonbattle injury was the result of a blast mechanism, and 57.7% of blast injuries resulted in concussion compared with only 4.6% of nonblast injuries. The lowest correlations were between ISS and year of injury (0.011), and ISS and age (0.031).

**Table 2:** Tetrachoric correlation matrix for covariates (*n* = 1179)

Variable	Age	Year of injury	Infantry	Battle injury	Blast injury	ISS	Concussion
Age	1.000	–	–	–	–	–	–
Year of injury	0.039	1.000	–	–	–	–	–
Infantry	−0.226	0.276	1.000	–	–	–	–
Battle injury	−0.141	0.357	0.683	1.000	–	–	–
Blast injury	−0.146	0.334	0.584	0.994	1.000	–	–
ISS	0.031	0.011	0.201	0.473	0.249	1.000	–
Concussion	−0.176	0.486	0.464	0.798	0.851	0.095	1.000

*ISS* Injury Severity Score

Table 3 details the results of univariate and multivariate logistic regression modeling. In univariate regression, all variables except for age were significantly associated with PTSD diagnosis (*p*s < 0.01). In multivariate modeling with backward selection, however, only battle injury (odds ratio [OR] = 6.64; 95% confidence interval [CI], 4.00–11.03) and bilateral hearing loss (OR = 2.92; 95% CI, 1.47–5.81) were significantly associated with PTSD. Unilateral hearing loss was not significantly associated with PTSD (OR = 1.23; 95% CI, 0.69–2.17). The Hosmer Lemeshow test had a *p*-value greater than 0.10, indicating a good model fit.

**Table 3:** Univariate and multivariate^a^ logistic regression analysis of hearing loss and PTSD (*n* = 1179)

Variable	PTSD	
	Univariate	Multivariate
	OR (95% CI)	OR (95% CI)
Hearing loss		
None	Ref	Ref
Unilateral	1.61 (0.93–2.81)	1.23 (0.69–2.17)
Bilateral	4.11 (2.13–7.93)^b^	2.92 (1.47–5.81)^b^
Age, years		NS
18–25	Ref	
26 and older	0.70 (0.42–1.17)	
Year of injury		NS
2004–2006	Ref	
2007–2012	1.57 (1.08–2.29)^b^	
Occupation		NS
Non-infantry	Ref	
Infantry	2.47 (1.66–3.67)^b^	
Battle injury		
No	Ref	Ref
Yes	7.07 (4.27–11.69)^b^	6.64 (4.00–11.03)^b^
Blast injury		NS
No	Ref	
Yes	4.66 (3.07–7.07)^b^	
ISS		NS
1–8	Ref	
9 and greater	2.86 (1.55–5.26)^b^	
Concussion		NS
No	Ref	
Yes	2.86 (1.96–4.19)^b^	

*CI* confidence interval, *ISS* Injury Severity Score, *NS* not selected, *OR* odds ratio, *PTSD* posttraumatic stress disorder, *Ref* reference level

^a^Multivariate model employed backward selection and was indicated as a good fit by the Hosmer-Lemeshow test (*p* > 0.10)

^b^*p* < 0.01

## Discussion

Hearing loss and PTSD are significant public health problems in the military. The present study suggests the two may be linked. In a large group of injured male military personnel, we found that the odds of PTSD are approximately three-times higher in individuals with postinjury bilateral hearing loss when compared to personnel without hearing loss. Hearing loss can affect listening performance and decrease quality of life, which may serve to exacerbate mental health problems such as PTSD [33].

Physical injury during combat is an established predictor of PTSD [23]. Koren et al. found that injured soldiers who experienced combat events similar to those of a noninjured control group were nearly nine-times more likely to develop PTSD [34]. Several studies have demonstrated that certain types of injuries may cause individuals to recall a traumatic event [35–37], including those with injuries that cause more severe physical problems, or impact appearance in daily life such as burns and facial trauma. It is possible that these injuries might exacerbate psychological effects of the traumatic event, thus increasing the chance and/or frequency of recollection, which in turn elevates the risk of PTSD. Extending this hypothesis, sensory injuries such as hearing loss can also adversely affect the life of working-aged adults; hearing loss acquired in adulthood has been found to lead to adjustment issues both personally and professionally [38]. It can result in mental distress due to social isolation, which can have a significant impact on personal relationships [39, 40]. In an occupational setting, workers with hearing loss report a greater sensitivity to background noise, communication issues that disrupt their work, and unsupportive supervisors [41, 42]. Injury-related hearing loss presents as an abrupt loss, which can leave the patient ill-prepared to deal with adjustment issues [43]. In summary, if an injury results in impairment that impacts daily life to a noticeable degree, whether that impairment is physical or sensory, the injury incident may be recalled more regularly, and thus resolution of the trauma might be more intractable. Studies using survey designs in clinical samples are needed to further explore this pathway.

Comorbidities of both PTSD and hearing loss may have played a role in the observed association. Depression has been frequently linked to PTSD and multiple studies have found increased rates of depression among those with hearing loss [33, 43, 44]. Abrams et al. found a strong correlation between hearing loss and depression in older veterans and concluded that individuals with both conditions had lowered measures of health-related quality of life [33]. Similarly, another study found that patients with abrupt or sudden hearing loss had a more than two times higher risk for developing depressive disorders than those without hearing loss [43]. Further, tinnitus commonly co-occurs with noise-induced hearing loss and has been associated with PTSD [45–47]. One study among a refugee population identified a significantly higher rate of PTSD among those with tinnitus and the study authors posited that tinnitus impacted trauma recall and flashbacks [46]. In addition, a study among military veterans found a correlation between PTSD and tinnitus, and reported that sounds triggering exacerbation of tinnitus similarly affected PTSD symptom severity [47]. Altogether, these findings indicate that longitudinal studies with multiple data ascertainment points are needed to investigate the effect of tinnitus and depression on the relationship between PTSD and hearing loss.

The present study has several strengths. In comparison with the recent study by Swan et al. that identified a relationship between PTSD and hearing loss using strictly diagnosis codes, the present study used audiometric data collected under standardized medical surveillance protocols [21]. The use of audiometric data to identify at-risk groups for PTSD and other mental health disorders has been investigated previously with mixed findings [48–50]. The results of this study may further encourage collaboration between audiologists and mental health professionals, particularly at military and VA hospitals where combat veterans are likely to be seen. The present study was also strengthened by the stratification of hearing loss into unilateral and bilateral, which added granularity to the results. In contrast to a previous study by Mahapatra that found no difference between bilateral and unilateral hearing loss with relation to depression, we found that those with bilateral hearing loss, but not unilateral, had a higher odds of PTSD [51]. Bilateral and unilateral hearing loss have different clinical profiles and further investigation is needed to determine what specific hearing difficulties (e.g., communication deficiencies, sound localization problems) have the greatest psychological impact [52]. Using the BRAID offers a unique examination of hearing loss in a population of mostly young adults. By using validated pre- and postinjury audiograms, we were able to capture hearing loss that occurred in proximity to the injury event. Last, the ability to link the BRAID to electronic medical records allowed for the abstraction of provider-diagnosed PTSD, thus avoiding the information bias issues found with self-reported data.

There are limitations that warrant mention. First, regarding the temporal relationship between hearing loss and PTSD, both were ascertained at different times postinjury. Using this methodology, it is possible that a diagnosis of PTSD might be recorded before or after the date of the audiogram and, as such, temporality could not be established. Using diagnosis codes to ascertain PTSD also excludes those individuals who may meet criteria for the disorder, but do not present for care. Moreover, a diagnosis of PTSD could be delayed by years, thus a diagnostic bias may occur with follow-up time restricted to two years. It is also possible that hearing loss could worsen between the blast injury and audiogram date, sometimes unrelated to the injury. A full assessment of combat experiences for each individual was not available for the present study. While it is arguable that a blast produced the hearing loss, it is likely that a collection of combat exposures caused the PTSD, the blast event being one. Though we accounted for injury-specific variables in our analysis, it may be difficult to fully adjust for the possible confounding effects of trauma severity on hearing loss. Additional controlled studies are needed to clarify these effects. Restricting the study sample to men precluded any examination of possible sex differences, and the findings may not generalize to military women. Lastly, potential comorbidities, such as tinnitus and depression, were not measured in the present study.

## Conclusions

To our knowledge, this is the first study to examine the association between PTSD and hearing loss in military personnel that have been injured during combat deployment. Taken together, both conditions are responsible for billions in health care costs for veterans. Delays in identification of these conditions may result in reduced quality of life for affected individuals, thus early provider diagnosis and intervention may be highly beneficial. Approximately 80% of primary care providers do not screen for hearing loss, and individuals with hearing loss often do not recognize a problem until they are examined and counseled [41]. This further encourages a strong working relationship between audiology and mental health professionals because military personnel with bilateral hearing loss should be monitored for possible development of PTSD. Future research should focus on determining etiological pathways and mediating factors, such as tinnitus and depression, and whether successful treatment of one condition has an impact on the other. Injuries of modern warfare can have serious audiological and psychological consequences, and health intervention strategies are paramount to maximize individual quality of life and overall military readiness.

## Data Availability

The data sets generated and/or analyzed during the current study are not publicly available due to personally identifiable information regulations, but may be made available by the corresponding author upon reasonable request and approval by the Naval Health Research Center Institutional Review Board.

## Abbreviations

BRAID: Blast-Related Auditory Injury Database
CI: confidence interval
DoD: Department of Defense
EMED: Expeditionary Medical Encounter Database
ISS: Injury Severity Score
MDR: Military Health System Data Repository
OR: odds ratio
PTSD: posttraumatic stress disorder
VA: Department of Veterans Affairs
